# Life History Parameters and Fishing Aspects of the Alien Nimble Spray Crab *Percnon gibbesi* in a Native Area of the Central-East Atlantic

**DOI:** 10.3390/ani13081427

**Published:** 2023-04-21

**Authors:** Airam Guerra-Marrero, Antonio Bonino-Pérez, Ana Espino-Ruano, Lorena Couce-Montero, David Jiménez-Alvarado, José J. Castro

**Affiliations:** IU-ECOAQUA, Universidad de Las Palmas de Gran Canaria, Edf. Ciencias Básicas, Campus de Tafira, Las Palmas de Gran Canaria, 35017 Las Palmas, Spain

**Keywords:** carapace length, length–weight relationships, ELEFAN, mortality, CPUE

## Abstract

**Simple Summary:**

We investigated the status and population structure of *Percnon gibbesi* in three areas of the island of Gran Canaria (Canary Islands, Central-East Atlantic) between July 2020 and December 2021. A total of 999 individuals were captured. Monthly sampling allowed us to establish the reproductive season according to the presence of ovigerous females. The methodology and number of individuals analysed were suitable for establishing the length–weight relationships and the life history parameters via modal progression analysis. The analysis of the catches per unit effort (CPUE) revealed the abundance of *Percnon gibbesi* in the three areas and seems to not conform with the catch quotas established in the Canary Islands Fisheries Law.

**Abstract:**

*Percnon gibbesi* is a native crab species characteristic of intertidal and subtidal zones of the Atlantic coast of the European Macaronesian archipelagos (Azores, Madeira, and Canary Islands), and probably also in the neighbouring rocky coasts of northwest Africa. *P. gibbesi* is considered an invasive alien species in almost all of the Mediterranean, with expanding populations from Spain to Turkey, including Libya; However, its biology and ecology are highly unknown, despite all its range of distribution. In the intertidal zones of Gran Canaria Island, this crab, in the intertidal zones of Gran Canaria Island, shows a carapace length range between 4.1 and 22.7 mm (4.1–22.7 in males and 5.7–22.3 in females), where females showed higher weights and lengths than males on average; However, males predominated in all samples, with a sex ratio of 1:0.57. The L_∞_ for this crab was estimated to be 27 ± 3 mm (23 ± 4 mm for females and 25 ± 4 mm for males). The growth coefficient (K) was 0.24 year^−1^, the total mortality (Z) was Z = 1.71 year^−1^, and the natural mortality (M) was 0.47 year^−1^_._ Although females grow faster than males, males are more abundant in the larger length classes. Although the presence of ovigerous females indicated that reproduction takes place twice a year, from March to April and from August to September, the number of cohorts detected by the modal progression analysis showed that reproduction takes place all year.

## 1. Introduction

Shellfishing on the intertidal zone for artisanal or recreational fishermen is a fairly important economic activity for human coastal populations worldwide [1]; however, this fishing activity is currently conducted on most coasts without control or supervision and is categorized as a data-poor fishery because there is not enough biological and fishing information on the target species to assess the status of the stock and establish reference points [2]. For the same reasons, in the Canary Islands, shellfishing is considered a data-poor fishery because professional and recreational shellfishers mainly exploit crabs that are, according to current legislation [3], used as bait to capture fish with higher economic value (e.g., *Sparisoma cretense*, [4]). 

Decapod crustaceans, particularly crabs, are of great fishing importance in relatively shallow waters of many parts of the world and support high-price markets [5,6,7,8,9,10,11,12]; however, crabs are also caught in subtidal zones targeted by spearfishers [13] or in intertidal strips through shellfishing on foot, where small-sized crab species predominate [14,15]. These other target species with a lower economic value are the mostly unknown regarding their biology and ecology. Even so, they play an essential role in the trophic flows of shallow ecosystems and the recycling of much of the organic matter produced in these areas [16,17].

Some studies have shown that crabs in benthic ecosystems often act as keystone species [18,19,20]. Many of these species are omnivorous, controlling the growth of algal species and small invertebrates, or recycling organic matter [21,22], so their lack or overexploitation can give rise to trophic cascades [16].

Overfishing, pollution, the introduction of foreign species, the alteration of coastal habitats, or changes in environmental conditions all negatively influence the functioning of shallow-water ecosystems, particularly in intertidal zones, increasing or decreasing the abundance of some crustacean species [18,23]. This may be the case for *Percnon gibbesi* [24], an autochthonous species from Atlantic waters, whose bioecology is almost unknown in its original distribution area and which is acting, possibly by finding favourable ecological and climatic conditions, as an invader in the Mediterranean [25].

The reproductive strategy of *Percnon gibbesi*, with a planktonic larval stage carried by currents, allows it to disperse over long distances [26]. Its routine habitat in the intertidal zone is usually on boulders that cover gravel bottoms and sands rich in organic matter, although its presence also extends along rocky infralittoral bottoms up to 25 m deep. Even so, *P. gibbesi* is more frequent in the intertidal zone closest to the limit of low tide. In the Canary Islands, it coexists with other species, such as *Pachygrapsus* spp., *Xantho* spp., or *Porcellana platycheles*, species that, like *P. gibbesi*, are used mainly as fishing bait. Even so, the target species in the Canary Islands’ shellfishing activity is *X. poressa*, with *P. gibbesi* being the by-catch. *Percnon gibbesi* has certain gregarious behaviours, and although it is of low density, it forms groups of two to five individuals [27].

*Percnon gibbesi* is a species with a wide geographic distribution, with a range that extends from Cabo San Lucas (Baja California) to Chile, including the Galapagos Islands in the eastern Pacific; from Fort Macon (North Carolina), Bahamas, and Bermuda to the Archipelago Fernando de Noronha in Brazil, including the West Indies in the western Atlantic; and from the Azores and Madeira to Angola in the eastern Atlantic [28]. Since 1999, it has also been present as an alien species in the Mediterranean Sea, initially reported off the island of Linosa (Strait of Sicily). The first sighting of the species in the Balearic Islands occurred in the early 2000s [29,30,31,32], expanding very rapidly since then [33] and currently considered an invasive alien species [34].

The lack of biological information on this species makes it vulnerable to uncontrolled extractive activity such as shellfishing. This study aimed to evaluate the status and population structure of *Percnon gibbesi* in three areas of Gran Canaria (Canary Islands, Central-Eastern Atlantic), defining its length–weight relationships and spawning season. Additionally, modal progression analysis was used to establish the life history parameters necessary for the correct management of the species. Finally, the catches per unit effort (CPUE) for the three areas were analysed to evaluate the shellfishing activity. Based on the UN Code of Conduct for Responsible Fisheries [35], this study seeks to contribute to the biological and ecological knowledge of *Percnon gibbesi* in the intertidal zones of the island of Gran Canaria, providing information that can be used to manage their populations—both in regions where it is native and where it acts as an opportunistic and invasive species—by regulating fishery and conservation plans.

## 2. Material and Methods

### 2.1. Study Area and Data Collection

**Figure 1:**
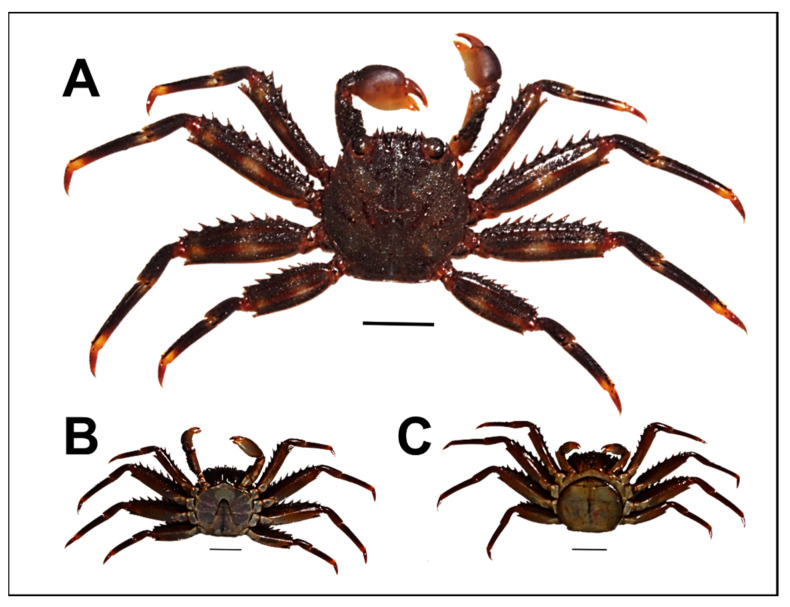
(**A**) Specimen of *Percnon gibbesi.* Male (**B**) and female (**C**) according to the abdomen shape. Scale bar = 1 cm.

**Figure 2:**
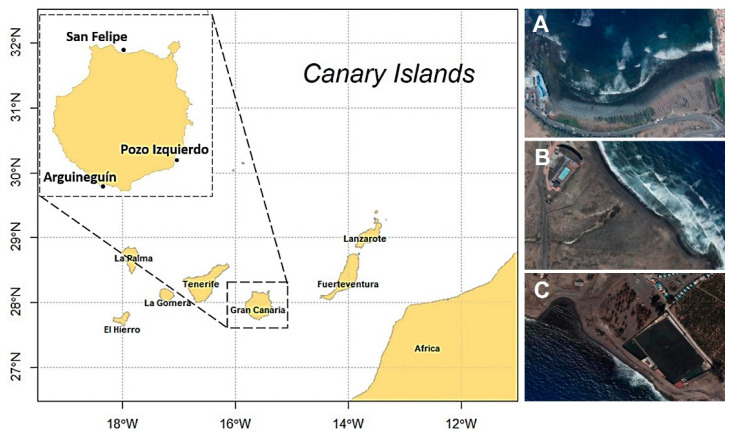
Location of the three sampled areas from Gran Canaria coast (Canary Islands, Spain). (**A**): San Felipe (north), (**B**): Pozo Izquierdo (east), (**C**): Arguineguín (south). Source (**A**–**C**): GRAFCAN, S.A. Canary Islands Government (2023).

Specimens of *Percnon gibbesi* (Figure 1) were caught monthly in intertidal zones of the coast of Gran Canaria (Canary Islands, Central-East Atlantic), between July 2020 and December 2021 (Figure 2). The intertidal zone was selected because this was where the shellfishing activity took place in the Canary Islands. Samplings were always conducted during low tide in three coastal localities of the island (San Felipe in the north, Pozo Izquierdo in the east, and Arguineguín in the south), taking all individuals (depletion method) found in three 1 m^2^ areas in each locality. Sampling areas of 1m^2^ were marked with a 1mx1m sampling quadrat, and the catches were made by hand, catching the crabs one by one. Throughout the study, 999 specimens were caught in order to determine the status and population structure of the species.

Carapace length (CL), carapace width (CW), and body weight (BW) were measured for each individual. The sex was identified according to the shape of the abdomen (triangular for males and rounded for females), and the females were classified as ovigerous or not ovigerous. All measurements and weights were taken with a digital calliper with a precision of 0.01 mm and a balance GRAM FV-220C with a precision of 0.0001 g.

### 2.2. Data Analysis

#### 2.2.1. Size Structure and Sex Ratio

Differences in CL distribution according to the months and area were investigated by applying the Shapiro–Wilk test using “stats” R package version 4.3.0 [36] to determine the normality of the data. Differences in the mean CL among months and area were tested with ANOVA and Wilcoxon post hoc test.

Proportions of males and females in each sampling area were compared with the expected 1:1 sex ratio using the goodness of fit chi-square tests (α = 0.05, [37]). The analysis was conducted using the “stats” R package [36].

#### 2.2.2. Length–Weight Relationships

The Length-Weight Relationship (LWR) was calculated by applying the power regression function BW = aCL^b^, where *a* and *b* are the regression parameters estimated by linear regression of the data logarithmically transformed and adjusted by the least squares method. Student’s *t*-test was used to verify the *b* values to determine whether they had isometric (*b* = 3) or allometric (negative allometric *b* < 3 and positive allometric *b* > 3) growth. CL–BW and CW–BW relationships were calculated separately for both sexes and areas, and the coefficient of determination (R^2^) obtained from this analysis was used as an indicator of the quality of the linear regression. The hypothesis of isometric growth [38] was tested using the *t*-test (*p* < 0.05).

#### 2.2.3. Growth and Mortality Parameters

The growth parameters were estimated through the von Bertalanffy growth function (VBGF) [39] using monthly CL frequency data (1 mm class interval). TropFish R package [40] was used to analyse the growth parameters according to the length frequency data with different optimisation techniques [41]. The asymptotic length and von Bertalanffy growth factor were calculated by the two optimisation approaches of ELEFAN: (i) ELEFAN with simulated annealing (ELEFAN SA) and (ii) ELEFAN with a genetic algorithm (ELEFAN GA). The ELEFAN model with the best scoring fit (high Rn) was selected. Bootstrap experiments for ELEFAN GA were based on 1000 resamples. The initial estimation of asymptotic length (L_∞_) was obtained from the Powell–Wetherall method [42].

TropFishR R package was used to estimate the total instantaneous mortality rate (Z) according to the length-converted catch curve analysis and the approximation of Pauly [43] using the length frequency data. The natural mortality (M), due to predation, senescence, or disease [44] was estimated by [45] approximation using the VBGF growth parameters (L_∞_ and k). The instantaneous fishing mortality rate, F, was deduced from the expression F = Z − M.

#### 2.2.4. Fishery Aspects

Catches per unit effort (CPUE; individuals and weight collected per hour^−1^ gatherer^−1^) was constructed to compare mean monthly catches (as a gross approximation to abundances) through the different areas. Welch’s heteroscedastic *F* test [46] was conducted to analyse CPUE differences between months (interannual variability) using the “misty” R package version 0.4.7 [47]. Analysis of Covariance (ANCOVA) was performed to detect CPUE differences between areas (months as the covariate variable), using the “car” package in R software [48]. A post hoc Tukey test was used to determine which areas differed significantly (using the “multcomp” R package, [49]).

## 3. Results

### 3.1. Size Structure and Sex Ratio

**Figure 3:**
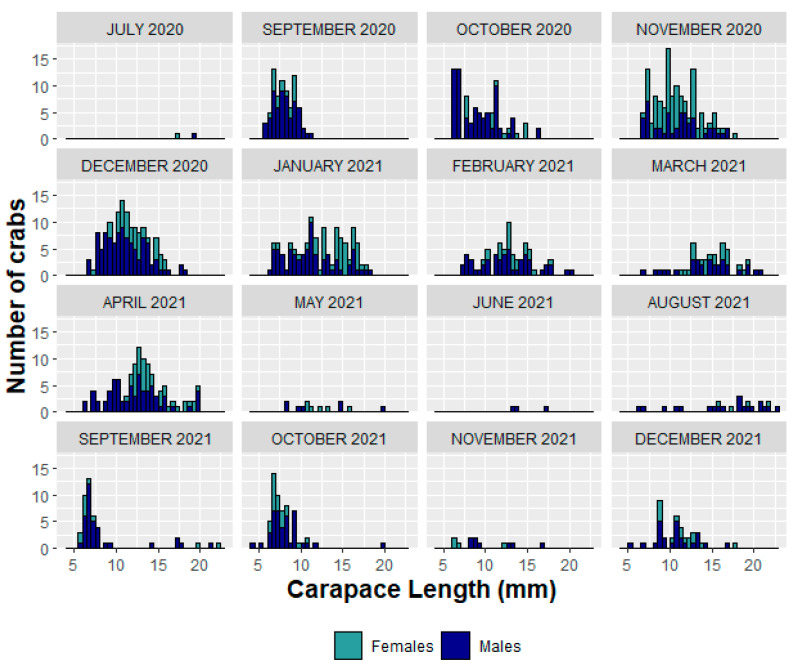
Monthly carapace length distribution of *Percnon gibbesi* caught in intertidal areas of Gran Canaria between July 2020 and December 2021.

A total of 349 females and 650 males were caught. The carapace length of Percnon gibbesi in the Canary waters ranged from 4.1 to 22.7 mm (4.1–22.7 [$\bar{X}$ = 10.78 ± 3.53] in males and 5.7–22.3 [$\bar{X}$ = 11.99 ± 3.30] in females; Figure 3). The highest number of individuals was captured in December 2020 (141 specimens), while in August 2020 and July 2021 no specimens were caught (Figure 3).

**Figure 4:**
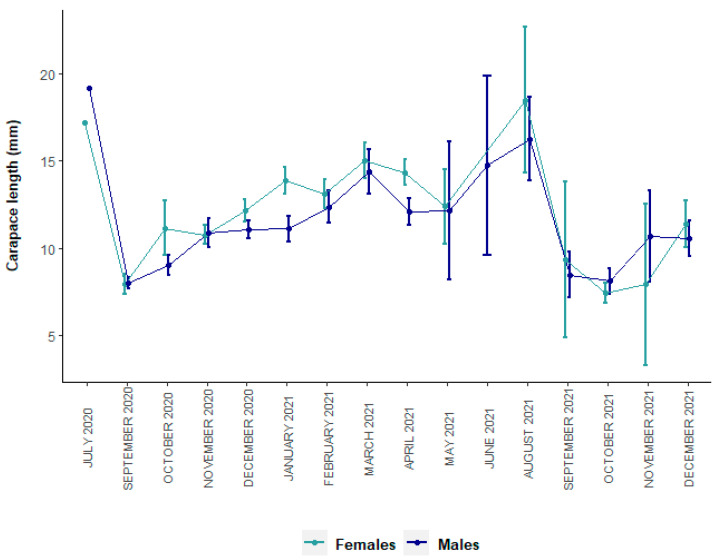
Monthly mean carapace length distribution, according to sex, in intertidal areas of Gran Canaria between July 2020 and December 2021.

No significant differences (ANOVA, *p* > 0.05) were found in the length distribution of crabs between the three sampled areas, but there were significant differences between sexes. The CL, CW, and BW show that, on average, female nimble spray crabs are heavier and larger than males (Table 1). The Wilcoxon test showed that females had higher CW (W = 99,029, N1 = 650, N2 = 349, *p* < 0.0001, Figure 4 and CL (W = 99,434, N1 = 650, N2 = 349, *p* <0.0001, Figure 4) than males. Similarly, females also had a higher BW compared to males (Wilcoxon test, W = 98,589, N1 = 650, N2 = 349, *p* < 0.0001).

For all areas, the mean length of crabs captured increased between September 2020 and August 2021, after which it dropped drastically, reducing the average length of individuals by more than 50% (Figure 4).

**Figure 5:**
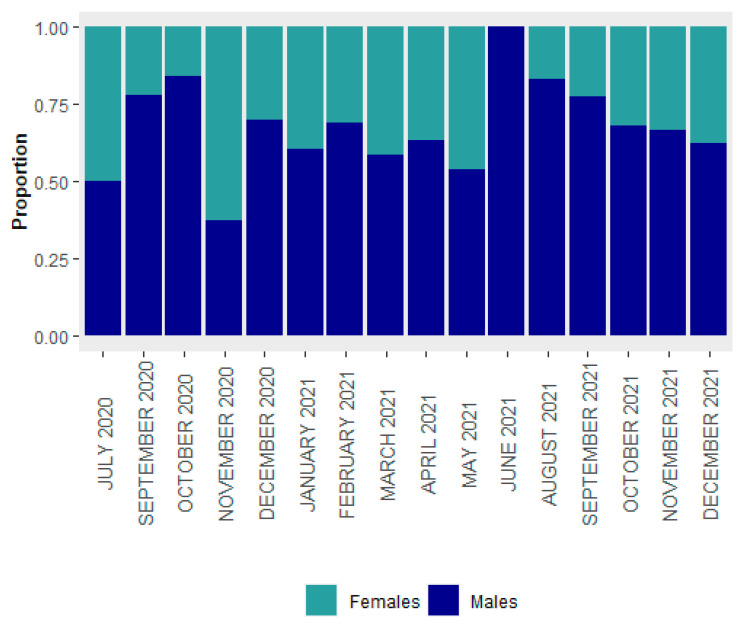
Proportion of *Percnon gibbesi* females and males caught in intertidal areas of Gran Canaria between July 2020 and December 2021.

**Figure 6:**
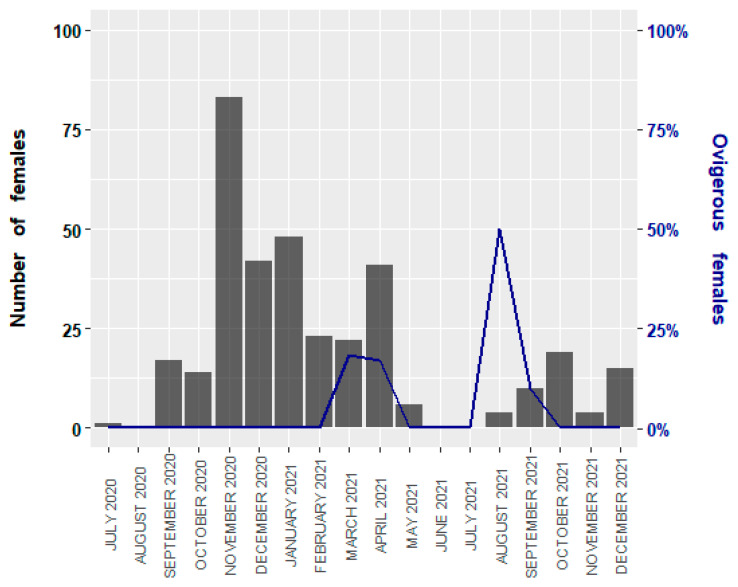
Percentage of ovigerous females (blue line) and the total number of captured females (grey bars) of *Percnon gibbesi* caught on the coast of Gran Canaria between July 2020 and December 2021.

Males predominated in the samples, with a global sex ratio of 1:0.57, but with significant differences between areas (Chi-Square test; *p*-value < 0.05) (Table 2). The proportion of males and females per month is shown in Figure 5. Females were only found in San Felipe in March, April, August, and September (Figure 6), but in very low quantities, being less than 20% of the total number of females captured in those months; however, in August 2021 about 50% of the specimens captured were female, because only four in total were caught that month.

### 3.2. Length–Weight Relationships

The length–weight relationships showed that *Percnon gibbesi* presents different growth models depending on the selected length measurement (Table 3). The entire population in relation to the CL showed a positive allometric growth, while in relation to the CW, it showed isometric growth. The length–weight relationships (CL–BW and CW–BW) did not present significant differences between areas (Kruskal–Wallis test, H = 8.67, *p* = 0.07), so individuals from the three areas could be considered from the same population.

### 3.3. Growth and Mortality Parameters

The length distribution varied greatly throughout the year. The mean length of the captured individuals was 11.20 mm (CL), whereas the length classes used in growth analysis with the ELEFAN methods ranged from 4 to 22 mm. The first L_∞_ estimations with the Powell–Wetherall method for the nimble spray crab was 27 ± 3 mm (23 ± 4 mm for females and 25 ± 4 mm for males), using this L_∞_ estimation in both ELEFAN methods. ELEFAN SA showed the best goodness of fit according to the ELEFAN GA method. The results of both methods for all populations are presented in Table 4.

The total mortality estimated from the catch curve was Z = 1.71 year^−1^. The natural mortality “M” obtained was 0.47 year^−1^ and the observed fishing mortality rate “F” was 1.24 year^−1^.

### 3.4. Fishery Aspects

**Figure 7:**
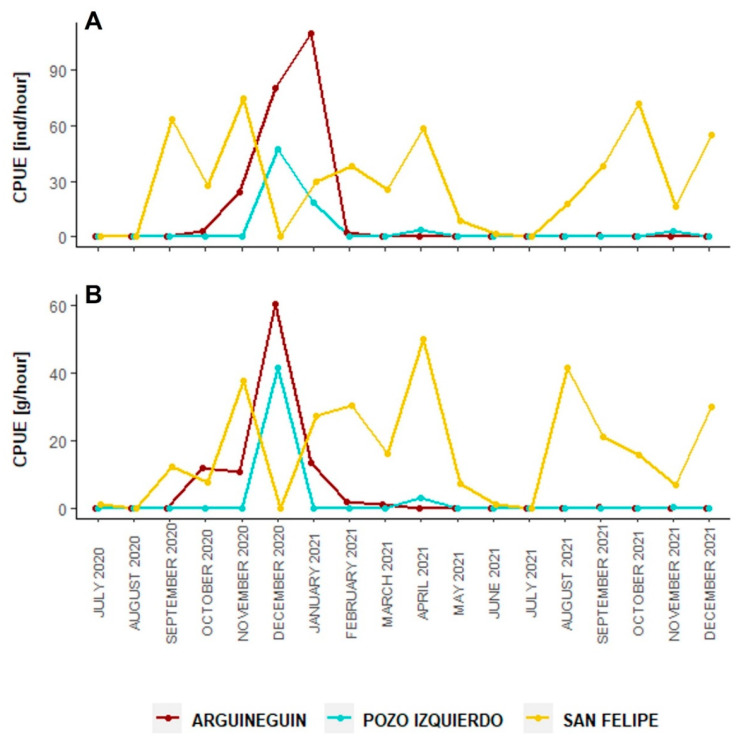
Mean catches per unit effort (CPUE), (**A**) CPUE, individuals per hour^−1^ gatherer^−1^; (**B**) CPUE, grams per hour^−1^ gatherer^−1^ recorded during 2020–2021 for *Percnon gibbesi* caught in three intertidal areas of Gran Canaria.

The CPUE showed an interannual variability (Welch’s test; F = 3.03, *p* = 0.0017) for all areas (Figure 7). In Arguineguín (south) and Pozo Izquierdo (east), the CPUE peaked in winter, while in San Felipe (north), it showed two peaks, one between late summer and autumn and the other between late winter and spring. The ANCOVA test of the CPUE showed a significant difference between areas (F = 8.177, *p* < 0.001). The post hoc Tukey test (t = 23.82, *p* < 0.05) determined that crabs from San Felipe showed significant differences with a higher CPUE (Biomass, Table 5).

## 4. Discussion

*Percnon gibbesi*, or the nimble spray crab, is a characteristic crustacean species of the intertidal and subtidal zones of waters in the East Atlantic [50] and has recently been classified as an invasive alien species in the Mediterranean Sea [34,51]. The number of months sampled was suitable to address the aims of this study, where the spawning season could be observed. In our study, we analysed the intertidal zones where *P. gibbesi* was distributed mainly on infralittoral rocky bottoms, although other authors have reported it at depths up to 25 m deep, on dense algae meadows, and in urchin-grazed barrens [52]. In this study, we corroborate that *P. gibbesi* coexists with other crab species, such as *Pachygrapsus* spp., *Xantho* spp., and *Porcellana platycheles* [50,53].

*Percnon gibbesi* in Gran Canaria intertidal zones shows a range of carapace lengths similar to those reported around other islands of the Canary Archipelago [54] but are slightly smaller than those described in different parts of the Mediterranean [27,33,55]. Males generally predominate, with a sex ratio of 1:0.57; but in the Mediterranean, females seem to be more abundant [27,56]. The authors reported a much higher proportion of ovigerous females in the Balearic Islands (87%) and in Sicily (96%) than in the Canary Islands, where this percentage does not reach 20% in those months that could be associated with peaks of the reproductive season (March to April and August to September). This difference may have several explanations, one of which is that we were sampling a small part of the population, and it is possible that a large part of it, including ovigerous females, was in subtidal areas where they were not accessible to our sampling. Ref. [27] carried out subtidal sampling up to 4 m deep, while for this work, we limited ourselves to the intertidal zone. *Percnon gibbesi* should also be considered as acting as an invasive species in the Mediterranean, in full expansion, while in the Canary Islands, it is an autochthonous species subjected to different ecological balances, and environmental, temperature, and food conditions that are less favourable for the species.

Similar to many crab species [57,58,59], males of *Percnon gibbesi* reach larger sizes than females, but females present an average carapace length larger than that of males. Although females grow faster than males, the latter are more abundant in the larger length classes. According to [60], this sexual dimorphism may be related to the role that each sex plays in the reproductive strategy of the species, which arises during the transition from juvenile to adult in the development of individuals. Thus, while in males the morphological changes are only a matter of carapace growth mainly in the frontal region, in females, it develops in different planes, possibly at the base of the abdomen to increase its capacity to house the spawn [61]; however, these variations in the growth pattern of males and females may be strongly conditioned by environmental variables of strictly local scope, particularly water temperature [62,63], but also due to fishing pressure [64]. In this sense, in Gran Canaria (depending on the sampling areas) males and females showed different growth patterns, where males showed an isometric and positive allometric growth, and the females showed isometric and negative allometric growth.

As previously indicated, the presence of ovigerous females was observed in two different periods—late winter–spring (March and April) and summer (August and September)—which may lead us to think that the species has two annual reproduction periods. Refs. [65,66] observed in Maltese waters that this species reproduces in summer, between May and September, and is recruited in winter, while ref. [56] established this reproductive period between July and November. Curiously, in Madeira, ref. [67] describe that the nimble spray crab reproduces in the month of April, which may indicate that environmental conditions largely determine the reproductive behaviour of the species [55]. However, the length frequency analysis done with the ELEFAN SA model detected that during the studied period (18 months) this crab population in Gran Canaria was composed of 12 cohorts. This high number of modal classes cannot be correlated with the months of the presence of ovigerous females in intertidal areas; instead, it indicates a large reproductive cycle that covers the entire year. In this sense, ref. [68] detected *Percnon gibbesi* larvae in zooplankton samples collected in the waters off Gran Canaria throughout the year, which indicates that the species has a continuous reproductive period; however, this is not reflected in the presence of ovigerous females in intertidal zones. In fact, ref. [68] describes two abundance peaks for nimble spray crab larvae, one in spring and another in summer.

The populations of *Percnon gibbesi* in the north, east and south of the island showed different dynamics. In the north, the species present several abundance peaks (mainly from January–April and August–October) throughout the year, the maximum values coinciding with the presence of ovigerous females. In the east and south it only presents a single peak in December. These results suggest the need to further identify if there is more than one stock of *Percnon gibbesi* on the island. The highest abundances of *Percnon gibbesi* were found on the north side of the Island, which almost duplicates that recorded on the south side, probably because it is a less fishable area due to an abrupt shore and sea conditions less favourable to gathering than in the south. According to current legislation [3], this species has a permitted quota of 500 g/day/gatherer for professional fishermen and 200 g/day/gatherer for recreational fishermen, which means that a gatherer needs to remove about 12 to 31 m^2^ of stones to reach the catch quota in the areas where this crab is more abundant, and about 24 to 61 m^2^ in the poorer area. The catch quotas for *P. gibbesi* populations in the current legislation seem to not conform to the estimated population size of this species, since, according to current recreational shellfishing licenses (29,013 licenses in 2020), the resource would be easily overexploited, as has been reported [4].

## 5. Conclusions

*Percnon gibbesi* captured on Gran Canaria Island showed an average carapace length of 11.2 mm, where females were larger on average. For the three study areas, males predominated over females, and two spawning peaks were established, one in late winter–spring (March and April) and another in summer (August and September), thanks to the presence of ovigerous females. In the modal progression analysis, ELEFAN SA was the best adapted for all the areas, where for all the individuals, an L_∞_ = 29.61, k = 0.24, M = 0.47, Z = 1.71 was obtained. The CPUEs showed a higher biomass in the north of the island for all months, except between December–January, when the greatest abundances were found in the east and south of the island. These data allow us to obtain information on the real and current state of these populations subjected to fishing exploitation. Having basic biological information will allow local administrations to establish measures for the sustainable management of this resource.

## Figures and Tables

**Table 1:** Summary of the values of carapace length (CL), carapace widths (CW) and total weights (BW) for males and females of *Percnon gibbesi* caught off Gran Canaria. N: number of individuals, Min: minimum, Max: maximum, SD: standard deviation.

			CW				CL				BW			
		N	Min	Max	Mean	SD	Min	Max	Mean	SD	Min	Max	Mean	SD
**Arguineguín**	**Males**	101	5.48	17.56	9.99	2.86	6.24	18.01	10.70	2.99	0.07	2.55	0.56	0.52
	**Females**	54	5.71	17.28	11.55	2.94	6.61	17.87	12.34	3.05	0.07	1.70	0.73	0.45
	**All**	155	5.48	17.56	10.53	2.97	6.24	18.01	11.27	3.10	0.07	2.55	0.62	0.50
**Pozo Izquierdo**	**Males**	37	6.65	14.76	10.38	2.16	6.88	15.36	10.98	2.24	0.15	2.19	0.58	0.42
	**Females**	11	5.92	14.95	10.74	2.43	6.85	15.45	11.47	2.21	0.12	2.01	0.67	0.50
	**All**	48	5.92	14.95	10.46	2.20	6.85	15.45	11.10	2.22	0.12	2.19	0.60	0.44
**San Felipe**	**Males**	512	3.15	21.08	9.94	3.57	4.15	22.73	10.78	3.70	0.01	5.19	0.64	0.79
	**Females**	284	5.19	19.96	11.04	3.23	5.69	22.30	19.95	3.39	0.06	3.55	0.74	0.60
	**All**	796	3.15	21.08	10.33	3.49	4.15	22.73	11.19	3.63	0.01	5.19	0.68	0.73
**All areas**	**Males**	650	3.15	21.08	9.97	3.40	4.15	22.73	10.78	3.53	0.01	5.19	0.63	0.74
	**Females**	349	5.19	19.96	11.11	3.16	5.69	22.30	11.99	3.30	0.06	3.55	0.74	0.58
	**All**	999	3.15	21.08	10.37	3.36	4.15	22.73	11.20	3.50	0.01	5.19	0.66	0.69

**Table 2:** Sex ratio of *Percnon gibbesi* caught in intertidal areas of Gran Canaria between July 2020 and December 2021.

	Sex-Ratio	Chi-Square	*p*-Value
Arguineguín	1:0.54	0.49	0.484
Pozo Izquierdo	1:0.27	6.25	0.012
San Felipe	1:0.60	17.94	<0.001
All areas	1:0.57	10.82	<0.001

**Table 3:** Length–weight relationships and growth model for males, females, and the entire population of *Percnon gibbesi* captured in intertidal areas of Gran Canaria (* highly significant, *p* < 0.0001) (i = isometric, a+ positive allometry, a− = negative allometry).

Relationships	Sex	a	b	CI 95%	CI 2.5%	R	*p*-Value	Growth Model
CL–BW	Females	0.0003	3.0042	2.9181	3.0903	0.9408	*	i
	Males	0.0002	3.1685	3.1103	3.2267	0.9566	*	a+
	All	0.0003	3.1085	3.0590	3.1570	0.9563	*	a+
CW–BW	Females	0.0005	2.9032	2.8169	2.9894	0.9368	*	a−
	Males	0.0004	3.0285	2.9681	3.0889	0.9492	*	i
	All	0.0005	2.9810	2.9300	3.3100	0.9501	*	i

**Table 4:** Growth parameters and scores obtained from ELEFAN SA and ELEFAN GA for females, males, and all population. The selected model was based on the highest Rn_max values, marked in bold.

Parameters	All Population		Females		Males	
	ELEFAN SA	ELEFAN GA	ELEFAN SA	ELEFAN GA	ELEFAN SA	ELEFAN GA
Asymptotic carapace length (mm)	29.61	26.76	28.11	25.17	28.56	25.36
Growth coefficient K (year^−1^)	0.24	0.34	0.22	0.33	0.19	0.28
Summer point oscillation (ts)	0.04	0.28	0.11	0.51	0.09	0.32
Amplitude of growth oscillation (C)	0.07	0.60	0.23	0.50	0.04	0.50
Growth performance index (Φ′)	2.03	2.21	2.24	2.26	2.19	2.14
t_anchor	0.56	0.74	0.26	0.71	0.41	0.51
Goodness of fit (Rn_max) score	**0.34**	0.31	**0.48**	0.35	**0.38**	0.36

t_anchor = time point anchoring growth curves in year-length coordinate system, corresponds to peak spawning month.

**Table 5:** Mean biomass captured and mean total biomass estimated of *Percnon gibbesi* for the three studied intertidal areas.

	Total Area (Ha)	Biomass Captured (g m^−2^)	Estimated Biomass Area (kg m^−2^)
Arguineguín	0.59	8.16 ± 5.06	48.14 ± 29.85
Pozo Izquierdo	0.45	10.65 ± 5.83	47.94 ± 26.25
San Felipe	0.63	15.06 ± 7.49	94.88 ± 47.19

## Data Availability

The datasets generated and/or analysed during the current study are available from the corresponding author upon reasonable request.
